# Diversity and Metabolic Potential of Gut Bacteria in *Dorcus hopei* (Coleoptera: Lucanidae): Influence of Fungus and Rotten Wood Diets

**DOI:** 10.3390/microorganisms13071692

**Published:** 2025-07-18

**Authors:** Pan Wang, Xiaoyan Bin, Xingjia Xiang, Xia Wan

**Affiliations:** 1 School of Resources and Environmental Engineering, Anhui University, Hefei 230601, China; wangp92621@163.com (P.W.); binxiaoyan10@163.com (X.B.); xjxiang@ahu.edu.cn (X.X.); 2 Anhui Province Key Laboratory of Wetland Ecosystem Protection and Restoration, Hefei 230601, China

**Keywords:** *Dorcus hopei*, gut microbiota, gut bacterial community, functional diversity, metabolic functions, diet

## Abstract

Stag beetles are saproxylic insects, essential for decomposing rotten wood and maintaining the carbon cycle. Their gut bacteria contribute significantly to nutrient digestion and energy acquisition, making them crucial for understanding host-microbe interactions. Despite the fungivorous behavior of stag beetle larvae, research on how diet influences gut bacterial diversity remains scarce. Therefore, this study was conducted to compare the diversity and metabolic functions of gut bacteria in *Dorcus hopei* larvae fed on fungus (*Pleurotus geesteranus*) and rotten wood diets using high-throughput sequencing technology. Significant differences (*p* < 0.05) were observed in gut bacterial community composition between two diets, highlighting diet as a key factor shaping bacterial diversity. Additionally, gut bacterial communities varied across larval developmental stages (*p* < 0.05), indicating the influence of host age. Dominant bacterial phyla included Firmicutes, Bacteroidetes, and Proteobacteria. Bacteroidetes were more abundant in rotten-wood-fed larvae (7.61%) than fungus-fed larvae (0.15%), while Proteobacteria were more abundant in fungus-fed larvae. Functional analysis revealed that rotten-wood-fed larvae were primarily related to carbohydrate and amino acid metabolism, whereas fungus-fed larvae exhibited enhanced membrane transport function. This study enhances the understanding of gut bacterial diversity and functions in stag beetles, providing a theoretical foundation for their conservation and sustainable utilization.

## 1. Introduction

Beetles, constituting the order Coleoptera, are one of the most diverse and species-rich groups within Insecta, accounting for approximately 40% of all insect species [1,2]. Through long-term evolution, they have developed complex and highly specialized gut systems [3]. A multitude of microbial communities colonize the insect gut, playing a critical role in aiding adaptation to complex environmental conditions and enabling the utilization of diverse food resources [4,5]. Within Coleoptera, Lucanidae species are notable for their pronounced sexual dimorphism and male trimorphism, with males possessing enlarged mandibles [6]. In the natural environment, larvae and adults exhibit distinct feeding behaviors: larvae inhabit and feed on rotten wood, while adults consume nectar or decaying fruit. Stag beetle, a representative species of decaying wood-feeding beetles [7], plays a vital role in decomposing dead wood and contributing to the carbon and nitrogen cycles in forest ecosystems. They are important contributors to forest health and occupy a distinct ecological niche [8]. Because stag beetle larvae feed on decaying wood, their gut microorganisms are crucial for lignin and cellulose degradation, enabling the digestion of this complex substrate [9]. Tanahashi first demonstrated fungivory in *Dorcus rectus* larvae for the first time by rearing on artificial diets containing mycelium from various wood-rotting fungi [10]. In fungal culture environments, insect gut bacteria act in concert with their fungal symbionts to influence the physiological functions of insects, providing critical support in areas such as metabolism and nutrition, and contributing to the maintenance of material cycling in the ecosystem [11].

As a widely distributed component of the insect gut microbiome, intestinal bacteria are essential for nutrient digestion, energy acquisition, and the overall health of their hosts [12]. Intestinal bacteria can produce various digestive enzymes to degrade complex carbohydrates in the diet and provide essential nutrients for insect growth [13]. They help insects overcome nutritional limitations posed by low-quality food sources [14]. In addition to aiding digestion, they play key roles in detoxification, strengthening immune defense [15,16,17]. Furthermore, gut bacteria enhance insect–microbe symbiotic relationships, influencing host development and promoting ecological adaptations. Salem and Kaltenpoth provided a comprehensive review of research on the gut bacteria of beetles, emphasizing the critical role of gut microbiome in beetle ecology [18]. The metabolic potential of microbial communities refers to the collective nutrient transformation and energy metabolism capacities encoded by their genomic metabolic pathways. Through this metabolic potential, microbial communities play pivotal roles in ecosystem functioning. Numerous studies have shown that diet strongly influences the structure and composition of the gut microbiota [12]. Different dietary components lead to changes in gut microbial diversity. For example, a study comparing gut bacterial abundance of wood-feeding beetles fed on different artificial diets found that diet significantly affects bacterial community composition [19]. This symbiotic relationship between beetles and their gut microbes enables diverse dietary adaptations [2]. Food sources introduce new microbial species or provide specific nutrients, altering the composition of the host microbiota [20]. A study has shown that dietary inclusion of defatted *Hermetia illucens* larvae meal (HM) in gilthead seabream (*Sparus aurata*) juveniles enhanced gut microbiota richness and diversity, while promoting the growth of potentially beneficial bacteria [21]. By comparing the gut microbiomes of silkworms reared on three distinct diets, the study discovered that the transitional diet group exhibited an intermediate gut microbiota complexity, characterized by specific bacterial taxa that may facilitate adaptation [22]. Research has shown that *Dorcus rectus* larvae contribute to nitrogen fixation, underscoring their ecological significance [23]. The gut microbiome of Cerambycidae species has a potential function in the degradation of lignocellulose [24]. Therefore, it is speculated that the gut bacteria of the stag beetles may also have a similar role.

To date, research on the diversity of gut microbial communities in stag beetles reared under artificial laboratory conditions has been limited. A study on *Phalacrognathus muelleri* has shown that the diversity of gut microbial communities varies significantly across developmental stages and sexes under artificial feeding conditions [25]. Similarly, the intestinal fungal community structure of *Dorcus hopei* was found to differ significantly among developmental stages under fungivorous conditions [26]. Research comparing the gut microbial composition and function of wild and domesticated *D. hopei* larvae revealed that diet and environmental conditions significantly influence bacterial community composition [27]. However, there is a paucity of studies on the effects of different diets (fungi and rotten wood) on the gut microbial diversity and composition of stag beetles under artificial rearing conditions.

*D. hopei*, a common saproxylic beetle widely distributed in China, is one of the largest Coleoptera species in East Asia [28]. This study focused on *D. hopei* larvae (the first, second, and third instars) reared on fungus-based and rotten-wood diets under laboratory conditions. We hypothesized that fungus-based and rotten-wood diets would differentially shape the composition and metabolic pathways of the gut microbiota in *D. hopei* larvae. High-throughput sequencing was employed to investigate the composition and metabolic potential of the larval gut bacterial communities under different feeding conditions, investigating how different diets affect the gut bacterial composition and the potential role of the gut microbiota in the growth and development of the stag beetle. Understanding these microbial interactions may have implications for stag beetle conservation and the development of optimized artificial rearing protocols.

## 2. Materials and Methods

### 2.1. Sample Collection and Rearing Conditions

A total of 79 larvae of *D. hopei* were purchased from the Mu-Ye Insect Company (Lishui, Zhejiang, China) and subsequently reared under controlled laboratory conditions on two distinct diets, namely, a rotten-wood diet (consisting of fermented wood chips that were compressed in a bottle) and a fungus-based diet (comprising bottled *Pleurotus geesteranus* mushroom packets). Both diets were provided by the Mu-Ye Insect Company. The larvae were maintained in an incubator under controlled artificial feeding conditions, with a temperature of 24 ± 2 °C, and the relative humidity maintained at 55 ± 5%. Regular monitoring of larval feeding behavior was conducted, with frequent replacement and hydration to prevent spoilage. In this study, the developmental stage of the larvae was determined by measuring the width of their head capsules. The head capsule diameter of the first instar larvae was approximately 3 mm, that of the second instar larvae was around 5 mm, and that of the third instar larvae was approximately 7–9 mm. Of the total samples, 40 larvae were fed on a rotten-wood diet (labeled as Larva_W), comprising the first instar (L1_W, 12 individuals), second instar (L2_W, 14 individuals), and third instar larvae (L3_W, 14 individuals). The remaining 39 larvae were fed on a fungus-based diet (labeled as Larva_F), comprising the first instar (L1_F, 13 individuals), second instar (L2_F, 13 individuals), and third instar larvae (L3_F, 13 individuals). After 15 days of feeding on the respective diets, the larvae were collected and underwent a two-day starvation period to eliminate transient food-associated microbes, so as to standardize gut contents. Following this, the samples were stored at −20 °C for subsequent dissection and experimental analyses.

### 2.2. Sample Dissection and Microbial DNA Extraction

Prior to dissection, the body surface of the *D. hopei* larvae was rinsed with distilled water, disinfected by soaking in 70% ethanol for three minutes, and subsequently washed twice with distilled water to remove residual ethanol and other contaminants [29]. Subsequently, the samples were washed twice with a 10-fold diluted phosphate-buffered saline (PBS) solution (total 500 mL containing NaCl 1.37 M, KCl 26.8 mM, Na_2_HPO_4_ 81.0 mM, KH_2_PO_4_ 17.6 mM, pH 7.2–7.4) to ensure thorough cleaning [30]. Dissections were performed in 10% PBS solution under a stereomicroscope (OLYMPUS, Tokyo, Japan) on a horizontal clean bench to maintain sterility. Using sterile fine-tipped forceps, the midgut and hindgut were carefully extracted and transferred into 2 mL Lysing Matrix E tubes under sterile conditions to prevent contamination [31]. Microbial DNA was extracted from the dissected gut tissues using the Fast DNA^®^ SPIN for Soil Kit (MP Biomedicals, Santa Ana, CA, USA) according to the provided protocol and stored at −20 °C for subsequent analysis.

### 2.3. PCR Amplification and 16S rRNA Sequencing

The purified DNA from each sample was used as the template for PCR amplification. The primer pairs 515F (5′-GTGCCAGCMGCCGCGG-3′) and 907R (5′-GGACTACHVGGGTWTCTAAT-3′) were employed to amplify the V4-V5 hypervariable regions of the bacterial 16S rRNA gene fragments [32]. The PCR reaction was performed in 20 μL reaction mixtures, including 10 μL of 2 × Pro Taq Master Mix, 0.8 µL each of forward and reverse primers, 10 ng of template DNA, and deionized water to make up the final reaction volume. The cycling parameters for PCR amplification program were as follows: an initial denaturation (at 95 °C for three minutes) and 29 cycles (of denaturation at 95 °C for 30 s, annealing at 53 °C for 30 s, and extension at 72 °C for 45 s) with a final extension (at 72 °C for 10 min). Negative controls without DNA templates were included to check for contamination. The amplification products were analyzed using 2% agarose gel electrophoresis to confirm product quality, and qualified amplified PCR products were sent to Majorbio (Shanghai, China) for subsequent sequencing. High-throughput sequencing was performed using the Illumina MiSeq platform (PE 300, San Diego, CA, USA).

### 2.4. Processing of Sequence Data

Raw bacterial data were initially processed using FLASH (v1.2.11) for preliminary analysis [33]. The resulting data were imported into the QIIME 2 pipeline (Quantitative Insights Into Microbial Ecology; qiime2-2023.5) for comprehensive processing [34]. Quality control was performed by filtering low-quality sequences using the ‘deblur’ algorithm [35,36]. The sequences were subsequently classified into amplicon sequence variants (ASVs). Chimera filtering was conducted using the VSEARCH (v2.13) method [37]. Sequence alignment was performed with the masked ‘MAFFT’ alignment tool [38], which removes highly variable regions to enhance analysis accuracy. The taxonomic assignment of ASVs was performed using the ‘classify-sklearn’ classifier with the Silva database (v138.1). Cluster analysis results were then utilized to examine species classification and community composition. To ensure comparability across samples, data were rarefied by randomly selecting 11,000 sequences (lowest-sequence-read depth) per sample with 20 repetitions to enable further comparison of bacterial community composition and diversity across all samples.

### 2.5. Statistical and Bioinformatics Analysis

Bacterial alpha diversity among dietary treatments was assessed using a one-way analysis of variance (ANOVA) with Tukey’s Honestly Significant Difference (HSD) post hoc testing in SPSS (v26.0, Chicago, IL, USA). The relative abundance of individual bacteria at the phylum and genus levels was calculated and visualized as stacked bar charts using the ggplot2 package in R (v4.3.2). Differences in larval gut microbiota structure were analyzed using a principal coordinates analysis (PCoA) and an analysis of similarities (ANOSIM; permutations = 999) using the vegan package in R (v4.3.2) [39]. Biomarkers of gut bacterial taxa with significant differences between dietary conditions were identified using linear discriminant analysis (LDA) effect size (LEfSe) [40]. The contribution of bacterial taxa to the differences between dietary conditions was analyzed by SIMPER analysis using the vegan package in R (v4.3.2) [41]. An indicator genera analysis was conducted using the labdsv package in R (v4.3.2) to identify bacterial taxa enriched under specific dietary conditions [42]. Predictions of bacterial community functions and pathways based on the 16S rRNA sequencing data were performed using the PICRUSt method, with the results matched to the KEGG database and visualized as bar plots grouped by dietary treatment using ggplot2 in R (v4.3.2) [43]. Co-occurrence network analyses of gut bacteria were performed using the dplyr, igraph, and Hmisc packages in R (v4.3.2) and visualized in Gephi (v0.9.2) [44], revealing interactions among bacterial taxa across dietary treatments.

## 3. Results

### 3.1. Larval Gut Bacterial Alpha Diversity

In this study, 15,868,850 high-quality bacterial sequences were obtained from 79 samples, with sequences per sample ranging from 11,716 to 38,079. A total of 3413 bacterial ASVs were identified, ranging from 26 to 798 ASVs per sample. A Venn diagram analysis revealed that 199 ASVs (5.8%) were exclusively found in the gut of Larva_F, 1333 ASVs (39.1%) were unique to the gut of Larva_W, and 1881 ASVs (55.1%) were shared between the two dietary groups (Figure S1C). This indicates that Larva_W harbored a greater number of unique bacterial ASVs compared to Larva_F. Unique ASVs also were identified at each larval developmental stage. Within Larva_F, 2080 ASVs were identified, of which 1141 ASVs (54.9%) were shared across all three instars, while distinct ASVs accounted for 4%, 6.6%, and 12.2% in the 1st, 2nd, and 3rd instars, respectively (Figure S1A). In Larva_W, 3214 ASVs were identified, with 1282 ASVs (39.9%) shared across all three instars, while the distinct ASVs accounted for 5.9%, 4%, and 21.9% in the 1st, 2nd, and 3rd instars, respectively (Figure S1B). These results indicate that the 3rd instar stage contained the highest proportion of unique intestinal bacterial ASVs under both dietary conditions.

**Figure 1:**
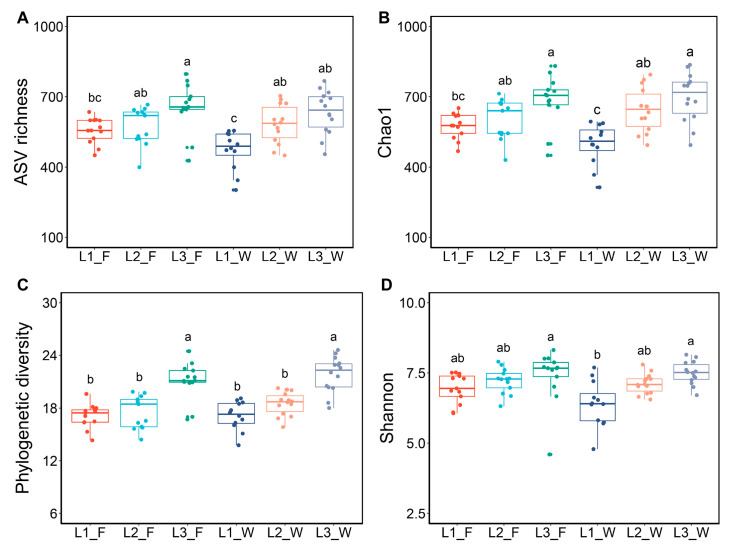
Intestinal bacterial alpha diversity in *D. hopei* larvae reared on two distinct artificial diets at different developmental stages. Diversity metrics include ASV richness (**A**), Chao1 (**B**), phylogenetic diversity (**C**), and Shannon index (**D**). Different letters above box plots represent significant differences according to one-way ANOVA (*p* < 0.05). L1_F, 1st instar larvae fed on fungus-based diet; L2_F, 2nd instar larvae fed on fungus-based diet; L3_F, 3rd instar larvae fed on fungus-based diet; L1_W, 1st instar larvae fed on rotten-wood diet; L2_W, 2nd instar larvae fed on rotten-wood diet; L3_W, 3rd instar larvae fed on rotten-wood diet.

In this study, we analyzed four alpha diversity indices, namely, ASV richness, Chao1, phylogenetic diversity, and the Shannon index to assess the variations in gut bacterial diversity across three developmental stages and between *D. hopei* larvae subjected to two distinct artificial diets. Bacterial alpha diversity exhibited a consistent trend across both dietary conditions, progressively increasing with larval developmental stages. The influence of host age on microbial diversity was significantly greater than that of dietary factors, with significant differences observed between the 1st and 3rd instar larvae (Figure 1). Additionally, the alpha diversity of Larva_F was marginally greater than that of Larva_W under both dietary conditions, although the differences were not statistically significant (Figure S2). The phylogenetic diversity index revealed that the 3rd instar larvae harbored a more diverse bacterial community. Notably, Larva_W exhibited greater phylogenetic diversity compared to Larva_F (Figure 1 and Figure S2C).

### 3.2. Intestinal Bacterial Community Structure

**Figure 2:**
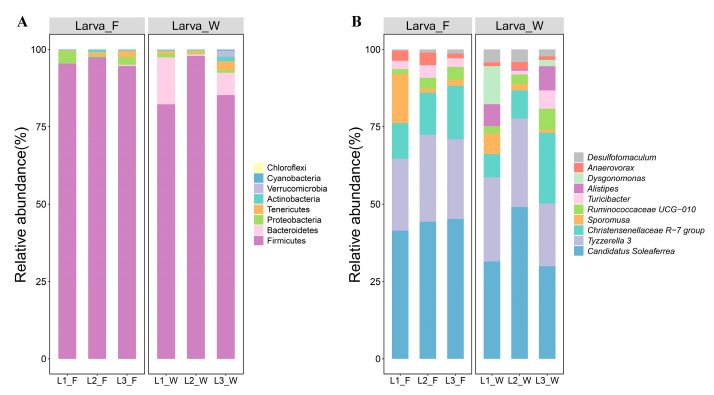
Comparison of the relative abundance of gut bacteria at the phylum level (**A**) and genus level (**B**) in *D. hopei* larvae reared on different diets. Larva_F, larvae fed on fungus-based diet; Larva_W, larvae fed on rotten-wood diet; L1_F, 1st instar larvae fed on fungus-based diet; L2_F, 2nd instar larvae fed on fungus-based diet; L3_F, 3rd instar larvae fed on fungus-based diet; L1_W, 1st instar larvae fed on rotten-wood diet; L2_W, 2nd instar larvae fed on rotten-wood diet; L3_W, 3rd instar larvae fed on rotten-wood diet.

The differences in gut bacterial community structure were the primary factors contributing to variations in beta diversity. An analysis of the bacterial community composition at the phylum level revealed that the dominant phyla (relative abundance > 1%) were consistent under both dietary conditions. These included Firmicutes (92.05%), Bacteroidetes (3.88%), and Proteobacteria (1.56%). Firmicutes exhibited the highest relative abundance in the larval gut across both diets, peaking in the 2nd instar larvae (97.71%). The relative abundance of Firmicutes in Larva_F (95.79%) was higher than that in Larva_W (88.31%). Comparatively, Bacteroidetes showed higher abundance in Larva_W (7.61%), with the highest levels observed in L1_W (15.16%). Conversely, Proteobacteria demonstrated greater abundance in Larva_F (2.25%), reaching their highest levels in L1_F (3.74%) (Figure 2A).

At the genus level, a total of 132 bacterial genera were identified in the larval gut. The dominant genera, with relative abundance exceeding 5%, included *Candidatus Soleaferrea* (25.01%), *Tyzzerella* 3 (15.79%), and the *Christensenellaceae* R-7 group (8.25%). Notably, the dominant bacterial genera under the two dietary conditions exhibited similar compositions and parallel trends in relative abundance changes. The relative abundance of *Candidatus Soleaferrea* (29.48%) and *Tyzzerella* 3 (17.84%) was highest in 2nd instar larvae, while the *Christensenellaceae* R-7 group gradually increased with larval age. However, trace amounts of *Alistipes* (2.96%) and *Dysgonomonas* (2.88%) were exclusively detected in Larva_W (Figure 2B).

**Figure 3:**
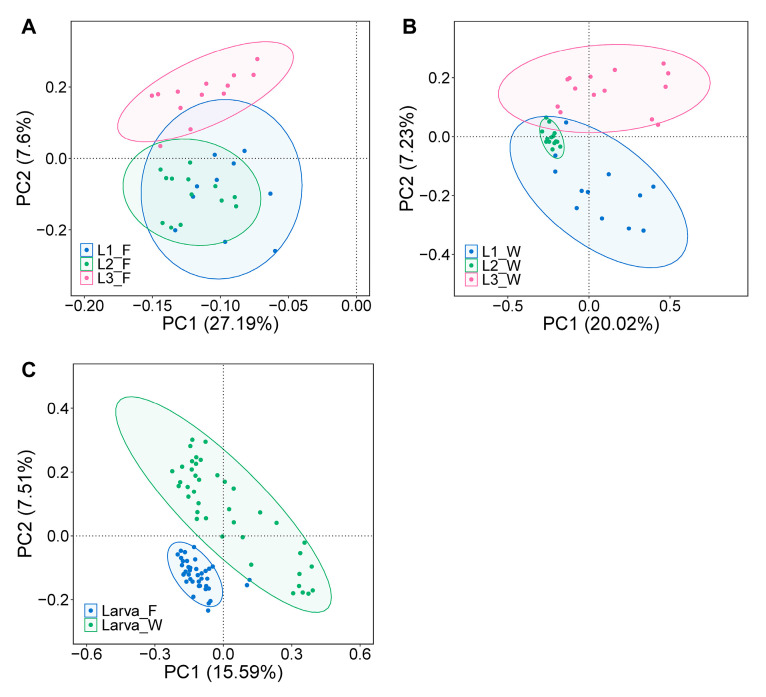
PCoA plots showing bacterial community composition (**A**) across different larval stages fed on fungus-based diet, (**B**) across different larval stages fed on rotten-wood diet, and (**C**) between larvae reared under the two dietary conditions. Larva_F, larvae fed on fungus-based diet; Larva_W, larvae fed on rotten-wood diet; L1_F, 1st instar larvae fed on fungus-based diet; L2_F, 2nd instar larvae fed on fungus-based diet; L3_F, 3rd instar larvae fed on fungus-based diet; L1_W, 1st instar larvae fed on rotten-wood diet; L2_W, 2nd instar larvae fed on rotten-wood diet; L3_W, 3rd instar larvae fed on rotten-wood diet.

The PCoA and ANOSIM analyses revealed significantly distinct gut bacterial community structures of *D. hopei* larvae across different larval instars and under two artificial diets. The PCoA scatter plot demonstrated that Larva_F samples were relatively more clustered compared to Larva_W samples. The two dietary groups were clearly separated, exhibiting highly significant differences in bacterial community composition (ANOSIM: *p* = 0.001; Table 1; Figure 3C). In Larva_F, the 3rd instar larvae were completely segregated from both the 1st instar larvae (*p* = 0.001) and 2nd instar larvae (*p* = 0.001), showing marked differences in community composition (Figure 3A; Table 1). Similarly, in Larva_W, the 1st, 2nd, and 3rd instar larvae were clearly segregated from each other, with significant differences observed in their bacterial community composition (*p* = 0.001, *p* = 0.003, and *p* = 0.001, respectively; Table 1; Figure 3B).

**Figure 4:**
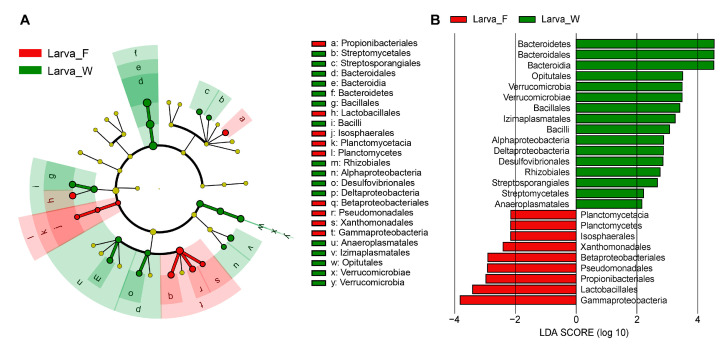
LEfSe analysis of gut bacteria in *D. hopei* larvae under two dietary conditions. (**A**) Cladogram illustrating the phylogenetic distribution of larval gut microbial communities under different diets. Yellow nodes represent microbial taxa with no significant difference between dietary groups, while other colored nodes represent microbial taxa significantly enriched in specific dietary groups. (**B**) Identified biomarkers ranked by effect size (LDA scores; alpha < 0.05) for each dietary group. Larva_F, larvae fed on fungus-based diet; Larva_W, larvae fed on rotten-wood diet.

The LEfSe analysis revealed specific bacterial taxa that were differentially abundant in *D. hopei* larvae under two dietary conditions. The cladogram illustrated the phylogenetic distribution of dominant bacterial taxa from the phylum to the order level in the two dietary groups (Figure 4A). The analysis focused on gut bacterial taxa with LDA scores greater than 2.0 (Figure 4B). Notably, nine bacterial taxa (one phylum, two classes, and six orders) were enriched in Larva_F, while 16 bacterial taxa (two phyla, five classes, and nine orders) were enriched in Larva_W (Figure 4).

The indicator genera with relative abundances exceeding 0.1% differed notably between the two dietary conditions, including 10 genera (e.g., *Candidatus Soleaferrea*, *Anaerovorax, Dendrosporobacter*) in Larva_F and 14 genera (e.g., *Dysgonomonas, Desulfotomaculum, Alistipes*) in Larva_W (Table S1). A SIMPER analysis revealed that *Candidatus Soleaferrea* was the main contributor to the differences in gut bacterial communities between Larva_F and Larva_W (Table 2).

### 3.3. Prediction of Gut Bacterial Function of D. hopei Larvae on Different Diets

**Figure 5:**
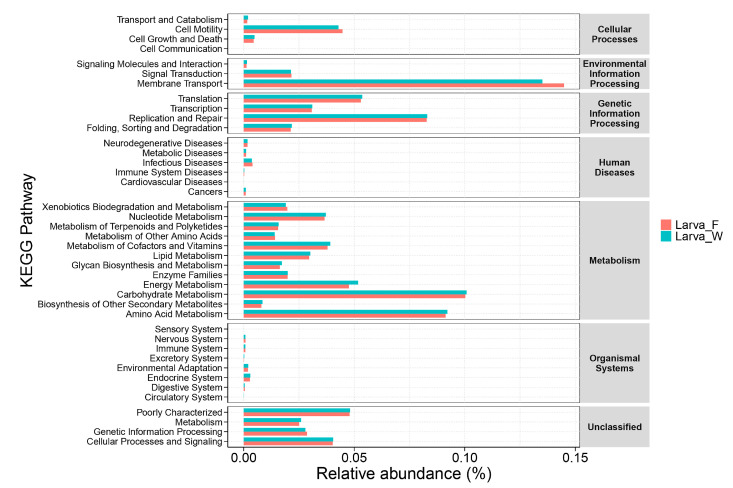
Relative abundance of KEGG pathways in gut genes of *D. hopei* larvae under different dietary conditions. Bar plots depict the relative abundance of each KEGG pathway, with the text on the right representing level 1 pathways and the text on the left indicating level 2 pathways. Larva_F, larvae fed on fungus-based diet; Larva_W, larvae fed on rotten-wood diet.

PICRUSt was employed to predict the potential functions of larval gut bacteria, revealing significant differences in microbiota functionalities between Larva_F and Larva_W. According to KEGG pathway annotation, the gut genes of *D. hopei* were categorized into six major functional categories: metabolism, genetic information processing, environmental information processing, cellular processes, organismal systems, and human diseases. Among these, the metabolism pathway (51.43%) represented the largest proportion of gene functions in both dietary groups. The relative abundance of metabolic pathways associated with gut genes was significantly higher in Larva_W (51.96%) compared to Larva_F. Within these metabolic pathways, the carbohydrate metabolism pathway was the most dominant, followed by amino acid metabolism, energy metabolism, metabolism of cofactors and vitamins, and nucleotide metabolism. In contrast, the membrane transport function was significantly enriched in Larva_F (14.40%) (Figure 5), highlighting distinct functional adaptations of the gut microbiota under different dietary conditions.

### 3.4. Network Analysis of Gut Bacteria in D. hopei Larvae on Different Diets

A co-occurrence network analysis was conducted to examine the relationships among gut bacterial communities in *D. hopei* larvae under two dietary conditions. The network diagram revealed that the predominant connections within the gut bacterial networks were associated with Firmicutes during the larval stage (Figure S3). The bacterial network of Larva_W exhibited a greater number of nodes and edges compared to Larva_F, indicating a higher degree of interaction within the gut bacterial community of Larva_W (Table S2). Additionally, the network structure of Larva_W showed a higher graph density, average degree, and average clustering coefficient, coupled with a shorter average path length and network diameter. These findings indicate that the gut bacterial community structure in Larva_W was more complex and compact than that in Larva_F.

## 4. Discussion

Diet has been established as one of the primary drivers influencing changes in the host gut microbial community [45]. Factors such as diet types, different developmental stages and host habitats significantly impact the structure of the gut microbiota [17,19,44]. The varying nutritional compositions in artificial rearing diets lead to synergistic coevolution between insects and their gut microbiota, enhancing their adaptive capacity to dietary changes and consequently influencing host development and physiological functions [46]. In this study, high-throughput sequencing was employed to analyze the gut bacterial communities of *D. hopei* larvae reared on two artificial diets: fermented wood chips and bottled *Pleurotus geesteranus* mushroom packets. This study represents the first comparative analysis of the gut microbial communities of *D. hopei* larvae fed on different artificial diets. The findings demonstrated significant differences in the gut bacterial communities between the two dietary conditions and across different larval developmental stages.

In this study, larvae fed on rotten-wood diet exhibited higher gut bacterial counts. Previous analysis of the nutritional composition of fermented wood chips in our laboratory revealed that they are rich in lignocellulose and hemicellulose, which likely promote bacterial proliferation and favor cellulolytic taxa [25]. Conversely, the bacterial content in the *Pleurotus geesteranus* fungus diet is generally low, which may explain the reduced gut bacterial counts in the larvae fed on this diet. The alpha diversity analysis showed no significant difference in gut bacterial diversity between the two dietary conditions. PCoA and ANOSIM analyses further revealed significant differences in the gut bacterial community structure between the two dietary conditions (Figure 3, Table 1). The SIMPER analysis identified *Candidatus Soleaferrea* as the primary factor contributing to the differences in gut bacterial communities between Larva_F and Larva_W (Table 2), suggesting that dietary composition may have an impact on shaping microbial communities. Additionally, differences across developmental stages were shown to significantly influence insect gut bacterial community composition, consistent with earlier findings [47]. During the rearing process, we observed a gradual increase in larval food consumption with developmental progression. The number and diversity of gut bacteria increased continuously with larval growth and development across the 1st, 2nd, and 3rd instar larvae, consistent with previous studies [25]. The gut bacterial communities of 3rd instar larvae were significantly distinct from those of 1st and 2nd instar larvae, likely due to increased food intake with larval growth and development. Notably, the 3rd instar larvae represent a critical period of rapid growth and development, characterized by higher nutrient demands. They need to obtain more nutrients and energy from food to prepare for the next stage of pupation. This increased dietary intake supports the colonization of a larger and more diverse microbial community, contributing to a richer gut bacterial ecosystem during this developmental stage.

The analysis of the bacterial community composition revealed that Firmicutes were the dominant phylum in larvae under both dietary conditions, followed by Bacteroidetes and Proteobacteria. This finding aligns with previous research on *D. hopei* [27] and other beetles, including *Phalacrognathus muelleri* [25], *Popillia japonica* [48], *Monochamus saltuarius* [49], and *Hylobius abietis* [50]. Firmicutes play a vital role in the degradation of plant carbohydrates, facilitating nutrient digestion and absorption in beetles. Notably, the relative abundance of Bacteroidetes was higher in Larva_W, compared to Larva_F, while the relative abundance of Proteobacteria was lower. These differences indicate that dietary components influence the proportion of dominant bacterial phyla and the balance of the overall gut microbiota composition. Proteobacteria are known to enhance nitrogen fixation and promote food metabolism [51], while Bacteroidetes can efficiently degrade complex polysaccharides (such as lignin and cellulose), potentially aiding the larvae in breaking down lignocellulose in rotten wood and promoting their growth [52]. At the genus level, *Candidatus Soleaferrea*, *Tyzzerella* 3, and the *Christensenellaceae* R-7 group were the dominant bacterial genera in both Larva_F and Larva_W. These genera are also enriched in the gut microbiome of wood-feeding beetles, such as Cerambycidae and Scolytidae larvae [53,54]. Furthermore, *Candidatus Soleaferrea* and *Tyzzerella* 3 were found to be core bacterial genera of *Macrotermes falciger*, which shares similar diets (wood and fungus feeding) with *D. hopei* larvae [55]. These dominant genera are all related to cellulose metabolism and plant-matter degradation, enhancing the larvae’s ability to degrade and utilize lignocellulose efficiently [52,56]. Additionally, *Alistipes* and *Dysgonomonas*, which were present in trace amounts in Larva_W, also make certain contributions to cellulose degradation and carbohydrate metabolism [57,58]. Their presence suggests they may work synergistically to improve degradation efficiency and enhance energy acquisition from woodchips in the larvae. Overall, the differences in dietary composition likely influence the dominant gut bacteria, leading to variations in the dominant bacteria present and their respective roles in nutrient processing.

Insects digest and absorb food in the gut, and their gut microbiota plays a crucial role in the transport and metabolism of carbohydrates and other nutrients. In this study, a PICRUSt analysis was employed to predict the potential functions of gut bacteria in *D. hopei* larvae fed on different diets. The gut gene functions were primarily associated with membrane transport and the metabolism of carbohydrates, amino acids, vitamins, and other nutrients, providing adequate nutrition and energy for the host. Compared to Larva_F, the gut gene functions of Larva_W were more enriched in metabolic pathways involved in carbohydrate metabolism, amino acid metabolism, and energy metabolism. This functional enrichment suggests that wood-feeding larvae may rely on microbial metabolism to compensate for the nutritional scarcity of diets rich in lignocellulose. Similar metabolic pathways are abundant in the gut bacteria of other wood-feeding beetles, where genes linked to lignocellulose degradation have been identified [24,56,59]. In the gut microbiota of *D. hopei* larvae fed on rotten-wood diet, the high abundance of Bacteroidetes is consistent with enhanced carbohydrate and amino acid metabolism functions. It is hypothesized that the components of woodchips may enhance the metabolic functions of gut microbiota, though this mechanism requires further investigation. Notably, the higher abundance of membrane transport genes in Larva_F may reflect a microbial strategy to maximize nutrient acquisition from a protein-rich fungal substrate. However, this hypothesis requires further validation through transcriptomic or metabolic flux analysis. The research on fungivorous beetles has revealed that beetles that feed on wood-rotting fungi require special digestive abilities to utilize the nutritional value of the fungal tissue [14]. The observed differences in food sources likely influence the absorption of gut nutrients, with larvae adapting to the unique dietary conditions by optimizing the gut nutrient uptake under fungal feeding conditions. However, further research with larger sample sizes is required to validate these findings.

The co-occurrence network analysis revealed that Firmicutes served as the key connecting cornerstone of the gut bacterial network, playing a pivotal role in structuring the community and facilitating specific beneficial functions (Figure S3). Network topological properties indicated that the bacterial network in the Larva_W network exhibited a higher clustering coefficient and shorter path length compared to Larva_F (Table S2). This suggests that the intestinal bacterial community of Larva_W possessed higher complexity and network stability, supporting more efficient material metabolism pathways, enhancing information exchange among its bacterial taxa and leading to greater collaborative capabilities [60]. Research has demonstrated that highly stable network structures exhibit greater resistance and adaptability to environmental fluctuations. Metrics such as the average path length and network diameter can reflect the responsiveness and resilience of microbial communities to environmental disturbances [61,62]. Compared to Larva_F, Larva_W demonstrated enhanced adaptability to environmental changes, and superior resistance and resilience to adverse conditions, facilitating the stability of their gut bacterial communities. These differences highlight the influence of dietary substrates on microbial ecology and suggest that gut microbiota may adaptively shift to optimize digestion under varying nutritional constraints.

## 5. Conclusions

In summary, this study elucidated the composition and diversity of the gut bacterial community in *D. hopei* larvae reared on different diets under artificial feeding conditions. The dominant phyla of gut bacteria in the larvae included Firmicutes, Bacteroidetes, and Proteobacteria. Significant differences were observed in gut bacterial community compositions between the two diets. The findings revealed that dietary differences significantly influenced the structure of the gut bacterial community, with distinct food sources leading to variations in the relative abundances of dominant bacterial taxa involved in food degradation. PICRUSt-based functional prediction suggested the enrichment in genes associated with carbohydrate and amino acid metabolism in wood-fed larvae, indicating microbial adaptation to lignocellulose-rich diets. These insights enhance our current understanding of the structure and function of the gut microbiome in stag beetles and highlight the potential function of gut microorganisms in host development. Additionally, the results provide a theoretical foundation for further research on the gut microbiota of stag beetles, the protection of saproxylic insects, and the potential utilization of insect-associated gut microbiomes.

However, this study has several limitations, such as the requirement to analyze the bacterial content of the diet in more detail to better understand its effect on the gut microbiota composition. Future research should employ metagenomic sequencing to explore the functional roles and metabolic processes of gut microorganisms in greater detail, and design experiments to verify the expression of genes related to lignocellulose degradation. Such studies could provide deeper insight into the interaction between microbial communities and their hosts through gene expression. These insights may pave the way for future advancements in insect microbiology and ecology.

## Figures and Tables

**Table 1:** ANOSIM results showing differences in gut bacterial community composition across larval stages and dietary conditions. Larva_F, larvae fed on fungus-based diet; Larva_W, larvae fed on rotten-wood diet; L1_F, 1st instar larvae fed on fungus-based diet; L2_F, 2nd instar larvae fed on fungus-based diet; L3_F, 3rd instar larvae fed on fungus-based diet; L1_W, 1st instar larvae fed on rotten-wood diet; L2_W, 2nd instar larvae fed on rotten-wood diet; L3_W, 3rd instar larvae fed on rotten-wood diet.

Stages	ANOSIM	
	*r*	*p*
L1_F vs. L2_F	0.129	0.023
L1_F vs. L3_F	0.272	0.001
L2_F vs. L3_F	0.350	0.001
L1_W vs. L2_W	0.324	0.001
L1_W vs. L3_W	0.251	0.003
L2_W vs. L3_W	0.356	0.001
Larva_F vs. Larva_W	0.211	0.001

**Table 2:** SIMPER analysis of gut bacterial composition in *D. hopei* larvae reared under different dietary conditions. Larva_F, larvae fed on fungus-based diet; Larva_W, larvae fed on rotten-wood diet.

Taxonomy	Contribution (%)
	Larva_F vs. Larva_W
g__*Candidatus Soleaferrea*	19.8
g__*Tyzzerella* 3	11.3
g__*Christensenellaceae* R-7 group	9.8
g__*Sporomusa*	9.2
g__*Alistipes*	5.3

## Data Availability

The raw data were submitted to the Sequence Read Archive (SRA) of NCBI under the accession number PRJNA1208448.

## Supplementary Materials

The following supporting information can be downloaded at: https://www.mdpi.com/article/10.3390/microorganisms13071692/s1, Figure S1: Venn diagram showing the co-occurrence of ASVs among larval samples fed on different diets. Numbers in parentheses represent the total ASVs identified in each group, while numbers within the Venn diagram indicate shared and unique ASVs. ASV, amplicon sequence variant. (A) Distribution of ASVs in larvae fed on fungus-based diets. (B) Distribution of ASVs in larvae fed on rotten-wood diet. (C) Comparison of ASVs between larvae fed on different artificial diets. Figure S2: Intestinal bacterial alpha diversity in *Dorcus hopei* larvae reared on two different artificial diets. Diversity metrics include ASV richness (A), Chao1 (B), phylogenetic diversity (C), and Shannon index (D). Figure S3: Co-occurrence network structure of gut bacterial community in *D. hopei* larvae under different dietary conditions. Table S1: Indicator genera with relative abundances > 0.1% in *D. hopei* larvae reared under different dietary conditions. Table S2: Topological properties of the co-occurrence network of the gut bacterial community in *D. hopei* larvae under different dietary conditions.
