# Supplementary material for: Diversity and Metabolic Potential of Gut Bacteria in Dorcus hopei (Coleoptera: Lucanidae): Influence of Fungus and Rotten Wood Diets
Source: Microorganisms. 2025 Jul 18;13(7):1692. doi: 10.3390/microorganisms13071692 (PMC12298515; doi:10.3390/microorganisms13071692)
Supplement: Supplementary file 1 [file microorganisms-13-01692-s001.zip › microorganisms-3687161-supplementary.pdf]

Supplementary Materials for

Diversity and metabolic potential of gut bacteria in *Dorcus hopei* (Coleoptera: Lucanidae): influence of fungus and rotten wood diets

Pan Wang [1,2], Xiaoyan Bin [1,2], Xingjia Xiang [1,2] and Xia Wan [1,2,*]

[1] School of Resources and Environmental Engineering, Anhui University, Hefei 230601, China; wangp92621@163.com (P.W.); binxiaoyan10@163.com (X.B.); xjxiang@ahu.edu.cn (X.X.)

[2] Anhui Province Key Laboratory of Wetland Ecosystem Protection and Restoration, Hefei 230601, China

* Correspondence: wanxia@ahu.edu.cn; Tel.: +86-13637091923

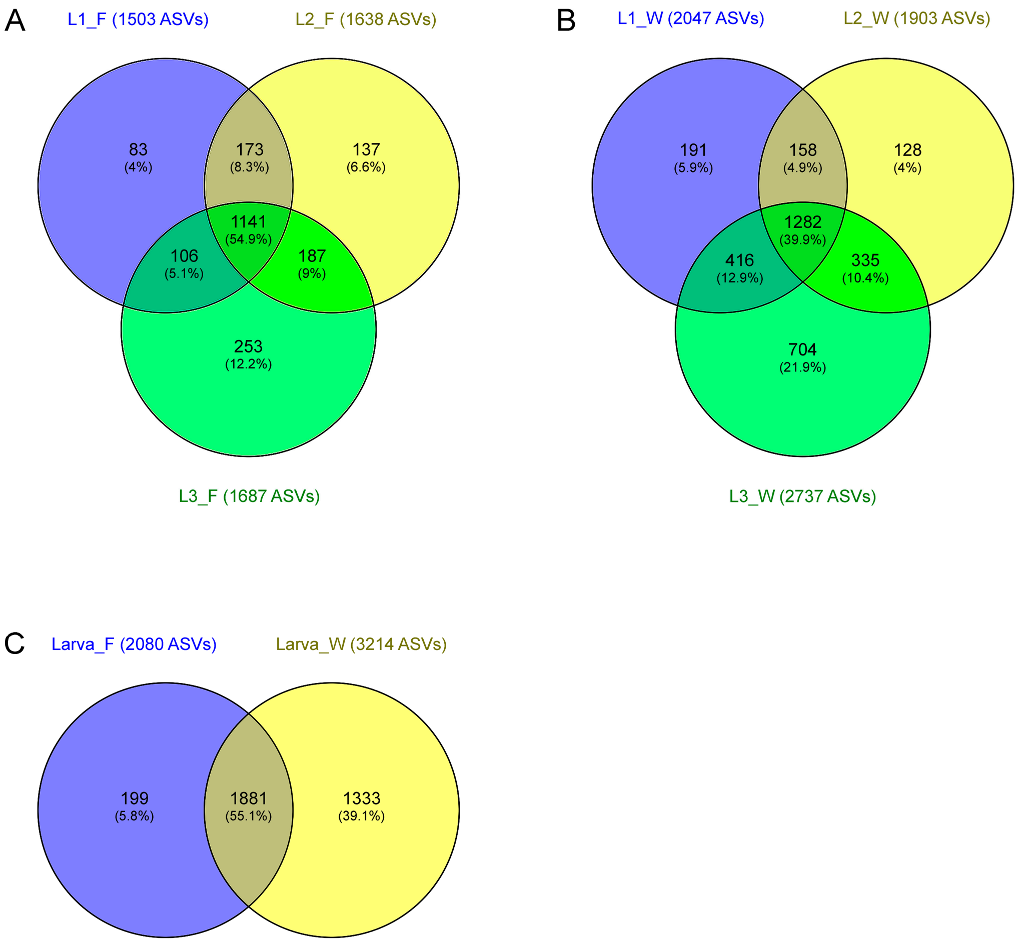

Figure S1. Venn diagram showing the co-occurrence of ASVs among larval samples fed on different diets. Numbers in parentheses represent the total ASVs identified in each group, while numbers within the Venn diagram indicate shared and unique ASVs. ASV, amplicon sequence variant. (A) Distribution of ASVs in larvae fed on fungus-based diets. (B) Distribution of ASVs in larvae fed on rotten-wood diet. (C) Comparison of ASVs between larvae fed on different artificial diet. Larva_F, larvae fed on fungus-based diet; Larva_W, larvae fed on rotten-wood diet; L1_F, 1st instar larvae fed on fungus-based diet; L2_F, 2nd instar larvae fed on fungus-based diet; L3_F, 3rd instar larvae fed on fungus-based diet; L1_W, 1st instar larvae fed on rotten-wood diet; L2_W, 2nd instar larvae fed on rotten-wood diet; L3_W, 3rd instar larvae fed on rotten-wood diet.

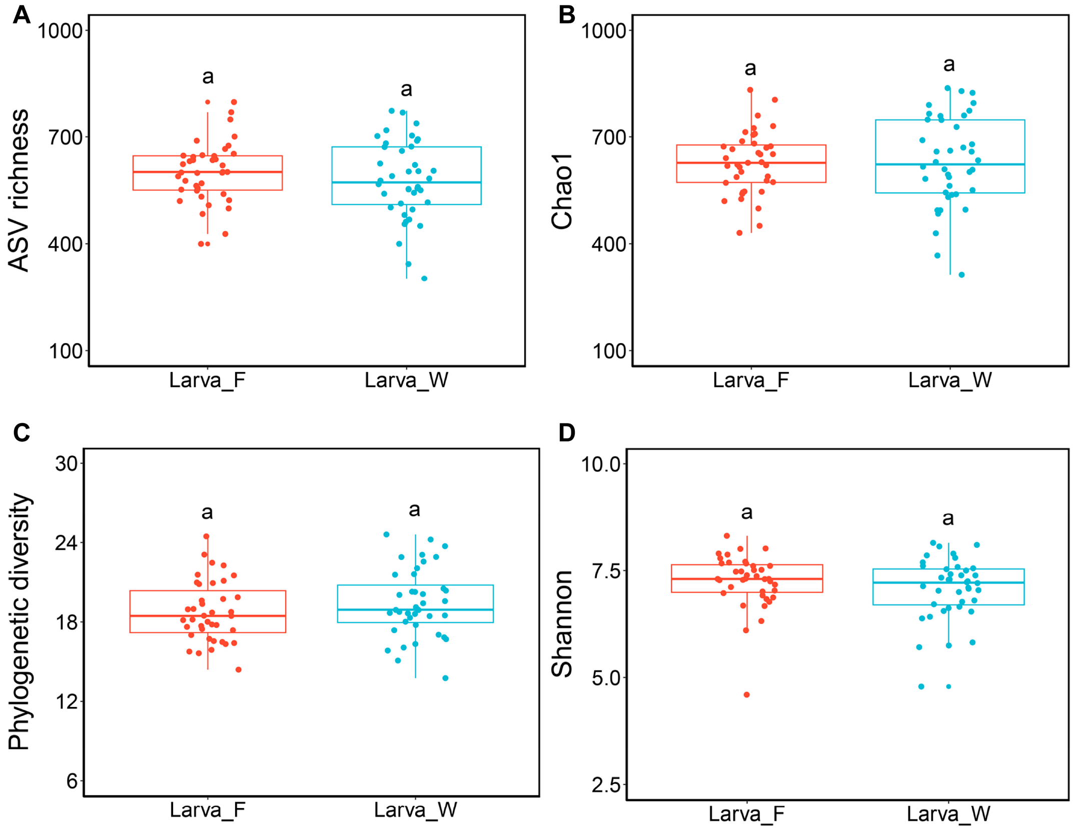

Figure S2. Intestinal bacterial alpha diversity in *Dorcus hopei* larvae reared on two different artificial diets. Diversity metrics include ASV richness (A), Chao1 (B), Phylogenetic diversity (C), and Shannon index (D). Different letters above box plots represent significant differences according to one-way ANOVA ($P < 0.05$). Larva_F, larvae fed on fungus-based diet; Larva_W, larvae fed on rotten-wood diet.

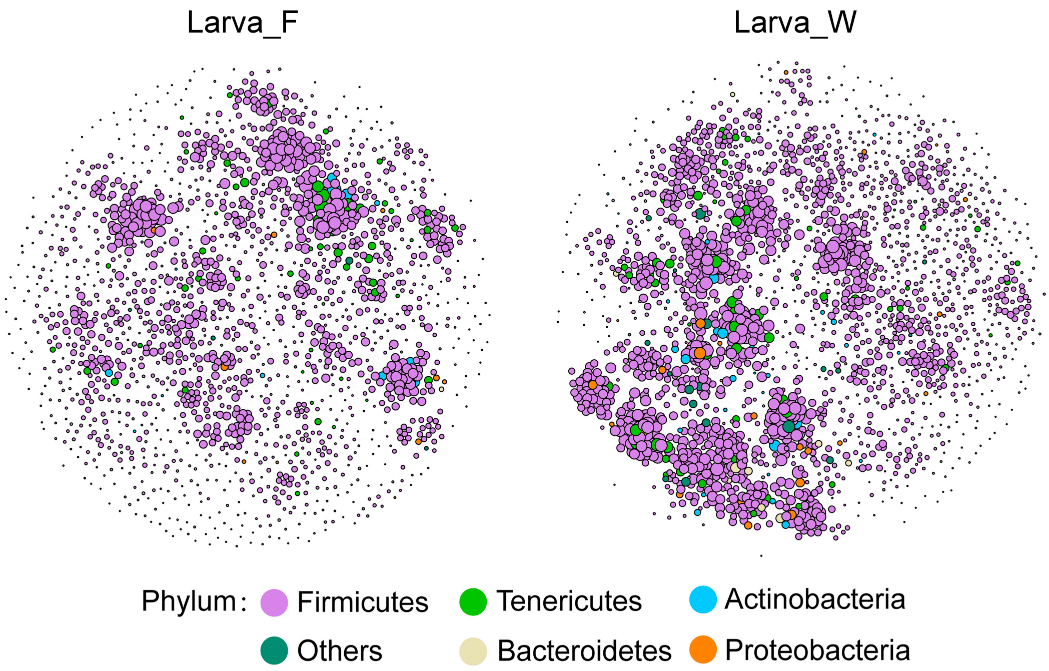

Figure S3. Co-occurrence network structure of gut bacterial community in *D. hopei* larvae under different dietary conditions. Larva_F, larvae fed on fungus-based diet; Larva_W, larvae fed on rotten-wood diet.

Table S1. Indicator genera with relative abundances > 0.1% in *D. hopei* larvae reared under different dietary conditions. Larva_F, larvae fed on fungus-based diet; Larva_W, larvae fed on rotten-wood diet.

**Stage**	**Taxonomy**	**Relative abundance (%)**	***P* value**
Larva_F	g__*Gryllotalpicola*	0.234	0.001
	g__*Anaerovorax*	2.563	0.024
	g__*Candidatus Dichloromethanomonas*	0.501	0.029
	g__*Anaerotruncus*	0.184	0.001
	g__*Candidatus Soleaferrea*	37.004	0.006
	g__*Intestinimonas*	0.410	0.004
	g__*Dendrosporobacter*	1.537	0.018
	g__*Burkholderia-Caballeronia-Paraburkholderia*	0.125	0.006
	g__*Oxalobacter*	0.453	0.001
	g__*Pseudomonas*	0.299	0.013
Larva_W	g__*Cellulomonas*	0.123	0.017
	g__*Dysgonomonas*	3.637	0.003
	g__*Alistipes*	3.982	0.001
	g__*Rikenella*	0.535	0.005
	g__*Bacillus*	0.598	0.001
	g__*Desulfotomaculum*	2.859	0.013
	g__*Papillibacter*	0.873	0.049
	g__*Ruminiclostridium* 1	0.482	0.001
	g__*Ruminococcaceae* UCG-009	1.413	0.041
	g__*Ruminococcaceae* UCG-013	1.214	0.001
	g__*Sporobacter*	0.201	0.019
	g__[Eubacterium] *coprostanoligenes* group	2.044	0.005
	g__*Desulfovibrio*	0.217	0.001
	g__*Diplosphaera*	0.912	0.001

Table S2. Topological properties of the co-occurrence network of gut bacterial community in *D. hopei* larvae under different dietary conditions. Larva_F, larvae fed on fungus-based diet; Larva_W, larvae fed on rotten-wood diet.

Network properties	Group	
	**Larva_F**	**Larva_W**
Node	1811	2216
Edge	45838	73476
Graph density	0.014	0.015
Network diameter	11	9
Average degree	25.311	33.157
Average path length	4.053	3.885
Average weighted degree	37.048	48.695
Average clustering coefficient	0.549	0.574
